# Responses to Increased Salinity and Severe Drought in the Eastern Iberian Endemic Species *Thalictrum maritimum* (Ranunculaceae), Threatened by Climate Change

**DOI:** 10.3390/plants9101251

**Published:** 2020-09-23

**Authors:** Sara González-Orenga, Calin Trif, Mͣ Pilar Donat-Torres, Josep V. Llinares, Francisco Collado, P. Pablo Ferrer-Gallego, Emilio Laguna, Monica Boscaiu, Oscar Vicente

**Affiliations:** 1 Mediterranean Agroforestry Institute (IAM), Universitat Politècnica de València, 46022 Valencia, Spain; sagonor@doctor.upv.es (S.G.-O.); jollipa@qim.upv.es (J.V.L.); mobosnea@eaf.upv.es (M.B.); 2 Institute for the Conservation and Improvement of Valencian Agrodiversity (COMAV), Universitat Politècnica de València, 46022 Valencia, Spain; trif@post.bgu.ac.il; 3 Department of Biotechnology Engineering, Faculty of Engineering Science, Ben-Gurion University of the Negev, 84105 Beer-Sheva, Israel; 4 Research Institute for Integrated Management of Coastal Areas (IGIC), Universitat Politècnica de València, Gandía, 46730 Valencia, Spain; mpdonat@eaf.upv.es; 5 Devesa-Albufera Service, Municipal Nurseries of El Saler, 46012 Valencia, Spain; fjcollado@valencia.es; 6 Valencian Centre for Forestry Research and Experimentation (CIEF)-Wildlife Service, Generalitat Valenciana, Quart de Poblet, 46930 Valencia, Spain; flora.cief@gva.es (P.P.F.-G.); laguna_emi@gva.es (E.L.); 7 VAERSA-Generalitat Valenciana, 46015 Valencia, Spain

**Keywords:** endangered species, water deficit, salt stress, halophytes, climate analysis, soil analysis, plant growth analysis, biochemical parameters, biodiversity, conservation programmes

## Abstract

*Thalictrum maritimum* is an endangered, endemic species in East Spain, growing in areas of relatively low salinity in littoral salt marshes. A regression of its populations and the number of individuals has been registered in the last decade. This study aimed at establishing the causes of this reduction using a multidisciplinary approach, including climatic, ecological, physiological and biochemical analyses. The climatic data indicated that there was a direct negative correlation between increased drought, especially during autumn, and the number of individuals censused in the area of study. The susceptibility of this species to water deficit was confirmed by the analysis of growth parameters upon a water deficit treatment applied under controlled greenhouse conditions, with the plants withstanding only 23 days of complete absence of irrigation. On the other hand, increased salinity does not seem to be a risk factor for this species, which behaves as a halophyte, tolerating in controlled treatments salinities much higher than those registered in its natural habitat. The most relevant mechanisms of salt tolerance in *T. maritimum* appear to be based on the control of ion transport, by (i) the active transport of toxic ions to the aerial parts of the plants at high external salinity—where they are presumably stored in the leaf vacuoles to avoid their deleterious effects in the cytosol, (ii) the maintenance of K^+^ concentrations in belowground and aboveground organs, despite the increase of Na^+^ levels, and (iii) the salt-induced accumulation of Ca^2+^, particularly in stems and leaves. This study provides useful information for the management of the conservation plans of this rare and endangered species.

## 1. Introduction

The study of plant responses to environmental abiotic stress conditions and stress tolerance mechanisms is one of the most active lines of research in plant biology, given its undoubted scientific interest and its practical implications in agriculture. These studies are also important to help design and implement conservation strategies for natural habitats of great ecological value, such as Mediterranean coastal salt marshes, which are highly threatened by human influence and highly sensitive to the effects of climate change [1,2,3]. The vegetation of these ecosystems includes different salt-tolerant species, or halophytes [4,5], some of them relatively abundant and present in many geographical areas, which constitute the typical salt marsh communities. Together with these common structural species, others, less frequent or even rare, are precisely those on which the uniqueness of each salt marsh depends and contribute substantially to increase the biodiversity of these specialised habitats. The coastal marshes near the city of Valencia, in eastern Spain, shelter a large number of such species of great ecological and conservation value, as many are endemic (including some exclusive Valencian endemics), or very rare, threatened or even extinct in recent times in part of the territory [6]. Most of these species have not been previously studied, and their limits and mechanisms of stress tolerance are virtually unknown.

*Thalictrum maritimum* Dufour (Ranunculaceae family), belongs to this category of species; it was identified by Jean-Marie Léon Dufour in 1860 in salt marshes near the city of Valencia, Spain [7,8]. This species is an Eastern Iberian endemic, growing only in a few coastal areas [9,10]. Its southern limit is located within the Albufera Natural Park, the most relevant protected area of the Valencian Community [11]. It is a perennial geophyte, with an erect stem up to 80 cm. The leaves are composed of small, lanceolate and narrow leaflets. The inflorescence is loose, and flowers have yellow petals, and numerous stamens, which exceed the corolla. The fruit is a spindle-shaped achene with fine ribs and a hard shell. It flowers from July to October [12], and fruiting takes place in September–October. The radius of dispersion of the seeds is not known. The accumulated seed germination under standard controlled conditions, only imbibed with distilled water, often does not reach more than 40% [13,14], but mechanical scarification and pre-treatments with sulphuric acid increase germination rate up to 80% [15].

The species is found worldwide only in four sites, all located in the Valencian Community. The species and its habitat are threatened by land degradation, agricultural and urban development, changes in the soil regime due to floods and increased salt levels, soil over-fertilisation, competition with invasive plant species, wildfires and touristic pressure [10]. The species is classified at the legal rank “Vulnerable” in the Valencian Catalogue of Endangered Flora Species [16] as well as the IUCN homonym rank in the Spanish Red List of Vascular Flora [16]. The populations are subjected to regular monitoring, and accessions of collected seeds are long-term stored in the Valencian Wild Flora Germplasm Bank, hosted by the CIEF (Valencian Centre for Forestry Research and Experimentation), a technical facility depending on the Valencian Wildlife Service—’Servicio de Vida Silvestre’, in Spanish. Because of its conservation interest, several institutions have developed propagation protocols, and population reinforcements and translocations have been performed [10,17]. However, despite these conservation efforts, many of the populations of this threatened endemic plant are in decline and the species has entirely disappeared from some locations over the last years (Servicio de Vida Silvestre, unpublished data and [18]). Regarding, for example, the total number of adult individuals present in the Natural Park of Albufera, it varied from 2406 plants in 2011, to 2516 in 2014 and 3333 in 2015, but decreased to 2381 in 2017 (Servicio de Vida Silvestre, unpublished data).

Unknown issues on *T. maritimum* behaviour include its tolerance limits to abiotic stress factors, as well as the mechanisms that enable this species to cope with a stressful environment. In addition to the usual factors of stress in saline habitats, salt marshes, as other wild habitats in the Mediterranean ecosystems, also suffered during the last decades from the effects of global warming, especially of increased temperatures in summer and prolonged drought throughout the year [3,19]. These effects of climate change will most likely also cause an increase in the salinity of these habitats in the years ahead, which could further affect the already threatened *T. maritimum* populations. The present study is based on a multidisciplinary approach, with the aims of analysing the: (i) habitat characteristics of populations of *Thalictrum maritimum* in the Natural Park of Albufera, (ii) climate analysis for the last 19 years, (iii) soil characteristics in areas where the natural populations are located, and in sites were future reintroductions are planned or proposed, (iv) limits of tolerance to drought and salinity of the species in greenhouse controlled conditions, and (v) the main mechanisms of response to abiotic stress by exploring the ion absorption and transport, synthesis of osmolytes and antioxidant responses of the plants, subjected to water deficit and salt stress treatments. The information provided by these analyses should explain the decline observed in *T. maritimum* populations over the last years, and help design and implement conservation programmes of this highly endangered species.

## 2. Results

### 2.1. Habitat Characteristics of the Thalictrum maritimum Populations in the Natural Park of Albufera

**Figure 1:**
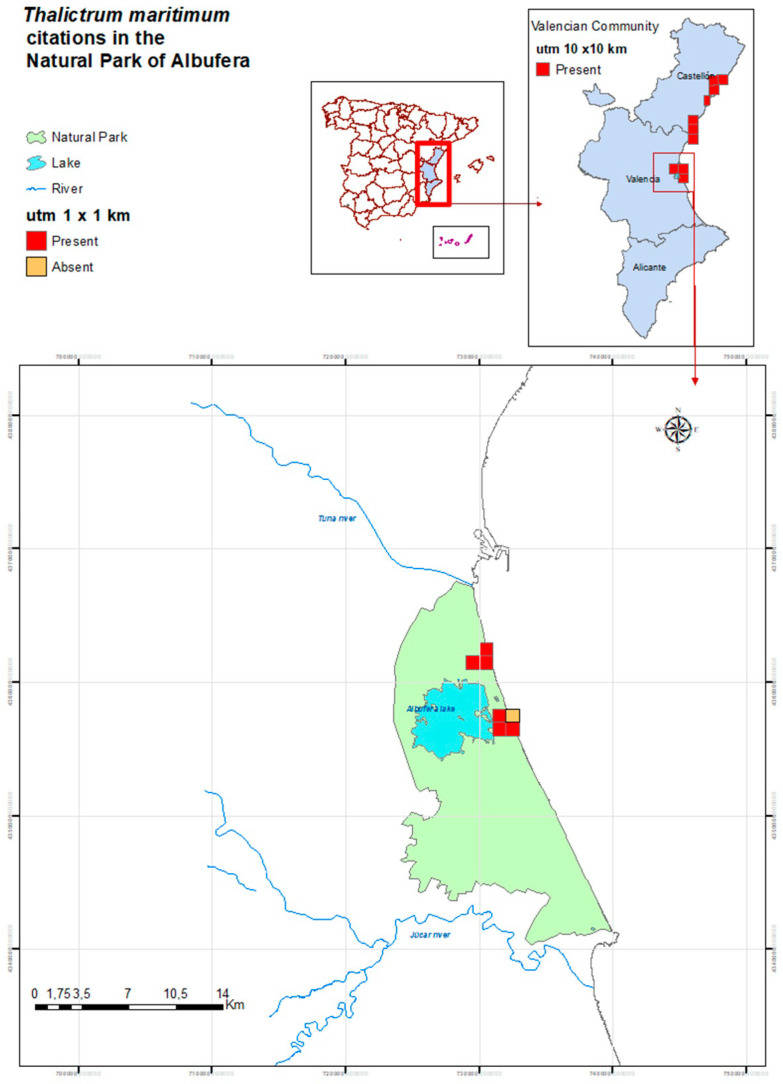
Location of the populations of *Thalictrum maritimum* in the Valencian Community (utm 10 × 10 km squares; upper right panel) and in the Natural Park of Albufera (utm 1 × 1 km squares; lower panel).

As a result of bibliographic searches and official consultations on the location of *T. maritimum* populations in the Natural Park of Albufera, a set of seven UTM 1 × 1 km squares was defined, corresponding to UTM30S YJ2961, 3061, 3062, 3156, 3157, 3256, 3257. However, after intense in situ surveys, only six of them hold current populations, having entirely vanished in the square 30S YJ3257, where the species lived in small salt marshes (Figure 1). 

The phytosociological study was performed during summer, coinciding with the optimal phenological period for *T. maritimum* and other species which grow on salt marshes. The species was found in ten locations in the natural park, but some of its previously reported populations no longer exist. Table 1 synthesises the habitat characteristics of each population (the extension and coverage of the plant community, soil moisture and electric conductivity), all species present in the community with their range of cover, according to Braun-Blanquet’s scale [20] and their corresponding vegetation classes [21,22].

The soil electric conductivity and moisture were measured with a WET sensor in each population, and values shown in Table 1 represent mean values of 8–10 measurements. Out of the ten analysed locations, only one area was truly saline (relevé 5, with an EC of 12.2 dS m^−1^), whereas in several soil samples EC was lower than 2 dS m^−1^. Soil moisture ranged from 40 to 60%. The highest presence of *T. maritimum* (4 in the Braun-Blanquet scale, representing a 50–75 % coverage) was found in the relevé 9, where soil salinity was the lowest (1.6 dS m^−1^)—except for relevé 7, with 1.3 dS m^−1^—and with 56% humidity. However, in relevé 3, where the soil EC was similar and humidity even higher, over 60%, only three individuals were detected (+ according to the scale). In the relevés 1, 2 and 4, *T. maritimum* had a good presence (3 on the scale, corresponding to 25–50 % coverage). Plant communities identified in each phytosociological relevé are indicated in Table 2. 

The relevés include some typical halophytic species, such as *Juncus acutus*, *J. maritimus*, *Plantago crassifolia* and *Schoenus nigricans*, but also many glycophytes, such as *Phillyrea angustifolia*, *Pistacia lentiscus* and *Smilax aspera* (species that characterise the vegetation class *Quercetea ilicis*). The presence of the invasive *Spartina patens* is remarkable, reaching high presence in relevés 5 and 7, and becoming a dominant species in number 6, shaping the plant community *Spartino-Juncetum maritimi* subass. *spartinetosum* O. Bolòs 1962. Other plant communities typical in the area were identified, belonging to *Juncetalia maritimi* Br.-Bl. ex Horvatic 1934, such as *Schoeno nigricantis*-*Plantaginetum crassifoliae* Br.-Bl. in Br.-Bl., Roussine & Nègre 1952, where the dominant species is *Juncus maritimus* (Cyperaceae family), and *Hydrocotylo-Mariscetum serrati* Rivas Goday & Mansanet.

### 2.2. Climate Analysis

Table 3 shows mean temperatures, mean maximal temperatures, mean minimal temperatures, mean atmospheric humidity, maximal and minimal humidity, rainfall and evapotranspiration obtained from the agroclimatological station Benifaio (at 11 km from the study area). According to the Worldwide Bioclimatic Classification System, 1996−2020 [23], these data match the thermomediterranean climate belt, specific for coastal and low-altitude areas, with a strong water deficit in summer, often with absolutely no rainfall for periods of one month or even longer. The evapotranspiration surpasses the rainfall amount during most months each year. 

**Figure 2:**
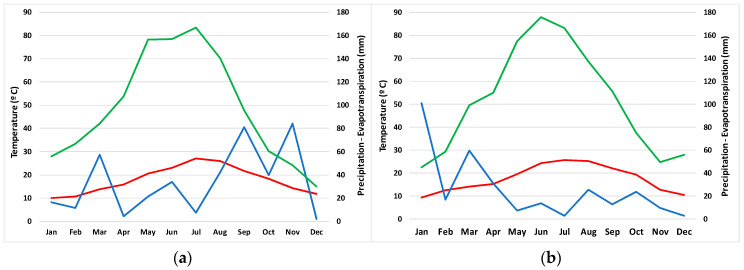
Climate charts including mean temperatures, rainfall and evapotranspiration during 2015 (**a**) and 2017 (**b**). Mean temperature is shown in red, rainfall in blue and evapotranspiration in green. Climatic data were from the nearest meteorological station in Benifaio, provided by the Agroclimatic Information System for Irrigation (SIAR) [24].

Climate charts indicating the evolution of mean temperatures, rainfall and evapotranspiration are shown for 2015 (Figure 2a), the year when the maximal number of individuals of *T. maritimum* was censused in the territory of the natural park, and 2017 (Figure 2b), when a drastic decrease in the number of individuals was registered, as mentioned in the Introduction. The main difference between the two years was the amount of rainfall, which dropped from 401.26 mm (close to the mean value for the 19 years) in 2015 to 307.26 mm (notably lower than the mean) in 2017 (Table 3). The distribution of the rainfall also differed in the two years; 2015 was characterised by a wet autumn (Figure 2a), contrary to 2017, when this season was exceptionally dry (Figure 2b).

### 2.3. Soil Characteristics

Soil characteristics were determined in the area where the most abundant population of *T. maritimum* was found in the Natural Park of Albufera, the salt marsh known as ‘Mallada del Canyar’. Soil samples were taken in the vicinity of plants, at two depths, 0–10 cm and 10–20 cm. Several physical and chemical soil parameters were measured and are summarised in Table 4. 

Soils have a sandy texture, the percentage of sand representing the major component, with low amounts of silt and clays. The pH is neutral, and the salinity at 0–10 cm depth is low. At 10–20 cm, depth, which is the area explored by the roots of the plants, the mean value of EC is below the limit for a soil to be considered as ‘saline’ (4 dS m^−1^). Amongst the analysed cations, Na^+^ is found at the highest concentration, almost double than that of Cl^−^. The soil samples are also characterised by a high percentage of carbonates, high concentrations of divalent cations, Ca^2+^ and Mg^2+^, and low K^+^ contents (Table 4). 

### 2.4. Substrate Electric Conductivity (EC) and Moisture

**Figure 3:**
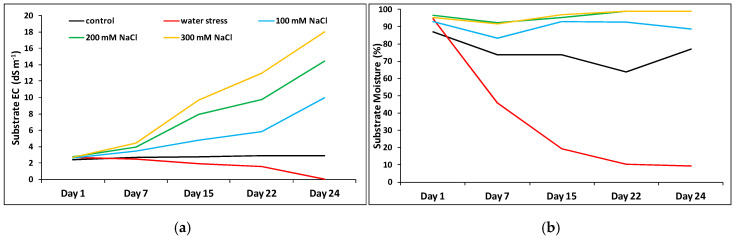
Substrate electric conductivity (dS m^−1^) (**a**) and moisture (%) (**b**) in the pots of the control and stressed plants of *Thalictrum maritimum* grown in controlled conditions in the greenhouse.

In the greenhouse experiments, substrate EC was measured throughout the treatments in all pots. As expected, it did not vary in the control treatment and even decreased slightly under water deficit conditions, but a marked time- and concentration-dependent increase was observed in the salt treatments, reaching a maximum of 18 dS m^−1^ in the pots watered for 23 days with 300 mM NaCl, due to the progressive accumulation of salt in the substrate (Figure 3a). Regarding the moisture of the substrate, a strong reduction was registered in the water stress treatment, which could be clearly observed already after one week of the absence of irrigation. Values of substrate moisture in the salt treatments were above those in the control, due to reduced absorption of water by the plants in the presence of salt (Figure 3b). 

### 2.5. Plant Growth under Stress in the Greenhouse

**Figure 4:**
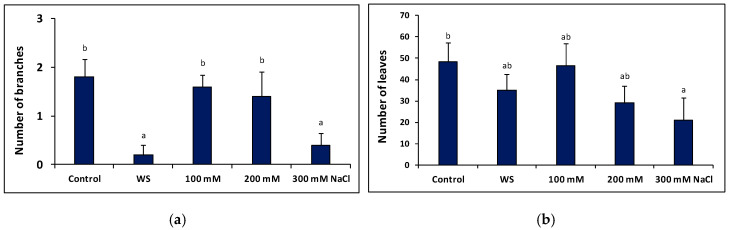
Number of new branches (**a**) and new leaves (**b**) formed during the 23 days of water stress (WS, complete withholding of irrigation) and salt stress (at the indicated NaCl concentrations) treatments, in *Thalictrum maritimum* plants grown under controlled conditions in the greenhouse. Values are calculated as the differences between the number of branches, or leaves, counted at the end and at the beginning of the corresponding treatments. Values shown are means ± SE (*n* = 5). Same lowercase letters over the bars indicate homogeneous groups between treatments according to the Tukey test (*p* ≤ 0.05).

Plants were subjected for 23 days to water deficit (completely stopping irrigation) and salt stress (100, 200 and 300 mM NaCl) treatments in the greenhouse. Determination of several morphological parameters indicated that both treatments caused inhibition of plant growth, in relation to the non-stressed controls, although in the case of salt stress a significant reduction of growth was only observed at high external salinities. For example, the number of new branches developed during the growth period, calculated as the difference between the last and first days of the treatments, decreased significantly in water-stressed plants. The mean values of new branches also decreased with increasing salinity, in a concentration-dependent manner, but the difference with the control was statistically significant only in the presence of the highest salt concentration tested, 300 mM NaCl (Figure 4a). The reduction on the average number of new leaves formed during the treatments showed a similar qualitative pattern as that of new branches but with quantitatively smaller differences with respect to the control, differences that were significant only for plants treated with 300 mM NaCl (Figure 4b).

**Figure 5:**
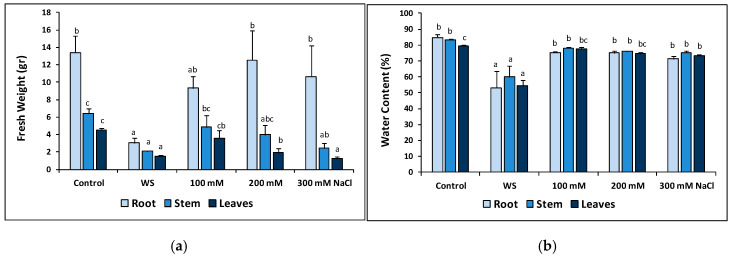
Fresh weight (**a**) and water content percentage (**b**) of roots, stems and leaves after 23 days of water stress (complete withholding of irrigation) and salt stress (at the indicated NaCl concentrations) treatments, in *Thalictrum maritimum* plants grown in controlled conditions in the greenhouse. Values shown are means ± SE (*n* = 5). For each organ, roots, stems or leaves, same lowercase letters over the bars indicate homogeneous groups between treatments according to the Tukey test (*p* ≤ 0.05).

The same strong effect of the water stress and the 300 mM NaCl treatments was also observed, in general, on the reduction of the fresh weight (FW) of the different organs of the plants (Figure 5a). The FW of roots, stems and leaves of water-stressed plants was significantly reduced as compared to the corresponding controls; for salt-treated plants, a decreasing trend with increasing salinity was also observed for the mean values of stem and leaf FW although, here again, a significant difference with the non-treated control plants was observed only at the highest salt concentrations tested (Figure 5a). The water content of roots, stems and leaves decreased significantly in the *T. maritimum* plants subjected to water deficit conditions, whereas this species seems to be highly resistant to salt-induced dehydration: a statistically significant (albeit slight) reduction in water content was only detected in the leaves of the plants grown in the presence of 300 mM NaCl, but not at lower salinities or in roots and stems at any tested salt concentration (Figure 5b). Root length did not show any significant variation in response to the stress treatments (data not shown). 

### 2.6. Photosynthetic Pigments

**Figure 6:**
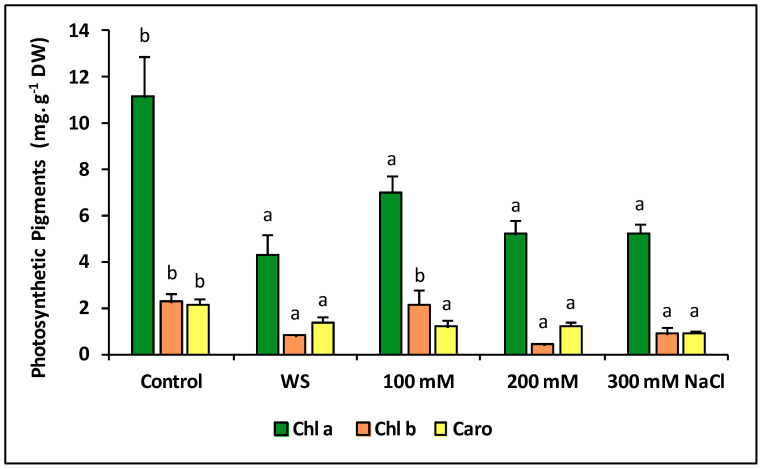
Photosynthetic pigments, chlorophyll a (Chl a), chlorophyll b (Chl b), and carotenoids (Caro) after 23 days of water stress (complete withholding of irrigation) and salt stress (at the indicated NaCl concentrations) treatments, in *Thalictrum maritimum* plants grown in controlled conditions in the greenhouse. Values shown are means ± SE (*n* = 5). For each pigment, same lowercase letters over the bars indicate homogeneous groups between treatments according to the Tukey test (*p* ≤ 0.05).

In combination with the determination of growth parameters, quantification of photosynthetic pigments (chlorophylls a and b, and carotenoids) is commonly used to assess the effects of stress on plants, as inhibition of photosynthesis, which is accompanied by a decrease in pigment contents, is generally observed under stress conditions. *Thalictrum maritimum* followed this general behaviour, as the concentrations of all photosynthetic pigments decreased significantly in response to water deficit and all salt treatments—except for chlorophyll b in plants treated with 100 mM NaCl, which showed similar values as in the control (Figure 6).

### 2.7. Ion Accumulation

**Figure 7:**
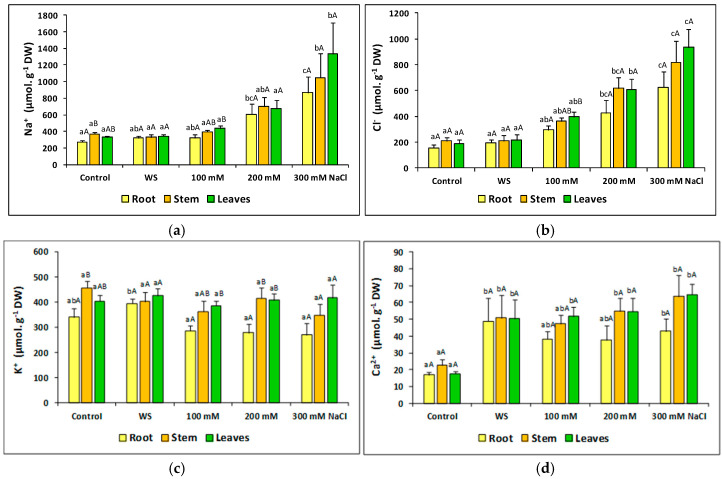
Ion contents, Na^+^ (**a**), Cl^-^ (**b**), K^+^ (**c**), Ca^2+^ (**d**), after 23 days of stress treatments, in roots, stems and leaves of *Thalictrum maritimum* plants grown in controlled conditions in the greenhouse. Values shown are means ± SE (*n* = 5). For each organ, same lowercase letters over the bars indicate homogeneous groups between treatments, whereas for each treatment same uppercase letters indicate homogeneous groups between roots, stems and leaves, according to the Tukey test (*p* ≤ 0.05).

As expected, Na^+^ and Cl^−^ contents did not show any significant variation in roots, stem or leaves of water-stressed plants, as compared to the corresponding controls (Figure 7a,b). The concentrations of the two ions increased in the presence of salt in the three organs, although the differences with the controls were not significant for the 100 mM NaCl treatment. For example, Na^+^ reached the maximum concentration (>1200 µmol g^−1^ DW) in leaves of plants treated with 300 mM NaCl, which represents a four-fold increase over control values (Figure 7a). The patterns of salt-induced variation in Cl^−^ contents were the same, although Cl^−^ concentrations were always somewhat lower than those of Na^+^ under the same conditions (Figure 7b). It is worth mentioning that both, Na^+^ and Cl^-^ accumulated at higher levels in the aboveground organs of the plants than in the roots, particularly at high external salinity (Figure 7a,b).

Regarding K^+^ contents, they did not vary significantly in response to the stress treatments in any of the assayed organs, and its levels were generally higher in stems and leaves than in the roots (Figure 7c). On the contrary, the water deficit and the salt treatments induced a notable increase in the concentration of Ca^2+^ in all organs of the plants, reaching two to three-fold higher values than the controls. As for Na^+^ and Cl^−^, Ca^2+^ levels were higher in the aerial part than in the roots of salt-treated plants (Figure 7d).

### 2.8. Osmolytes, Oxidative Stress Markers and Antioxidants 

Proline (Pro) and total soluble sugars (TSS) are common osmolytes in plants. Leaf concentrations of Pro increased in response to water deficit and salt stress. Still, both, the relative increment over control values (less than two-fold) and the absolute concentrations reached (about 20 µmol g^−1^ DW) were low. Leaf TSS contents decreased in water-stressed plants but did not vary significantly in those subjected to the salt treatments (Table 5).

Mean values of malondialdehyde (MDA), used as a marker of oxidative stress, and of representative antioxidant compounds, total phenolic compounds (TFC) and total flavonoids (TF), were lower in plants subjected to the water stress treatment than in the controls, but the differences with the non-stressed controls were not statistically significant. Concentrations of these compounds also showed a general decreasing trend with increasing salinity, although significant differences with the corresponding controls were observed only for TF at all salt concentrations, and for TPC in the presence of 300 mM NaCl (Table 5).

Regarding the specific activity of antioxidant enzymes, superoxide dismutase (SOD) increased significantly in the water stress treatment, but catalase (CAT) and glutathione reductase (GR) did not vary. In response to salt stress, CAT increased in the presence of 300 mM NaCl and GR in plants treated with 200 mM NaCl, whereas SOD did not show any significant variation (Table 5). 

### 2.9. Principal Component Analysis of Morphological and Biochemical Parameters Measured in Plants Grown under Experimental Greenhouse Conditions

**Figure 8:**
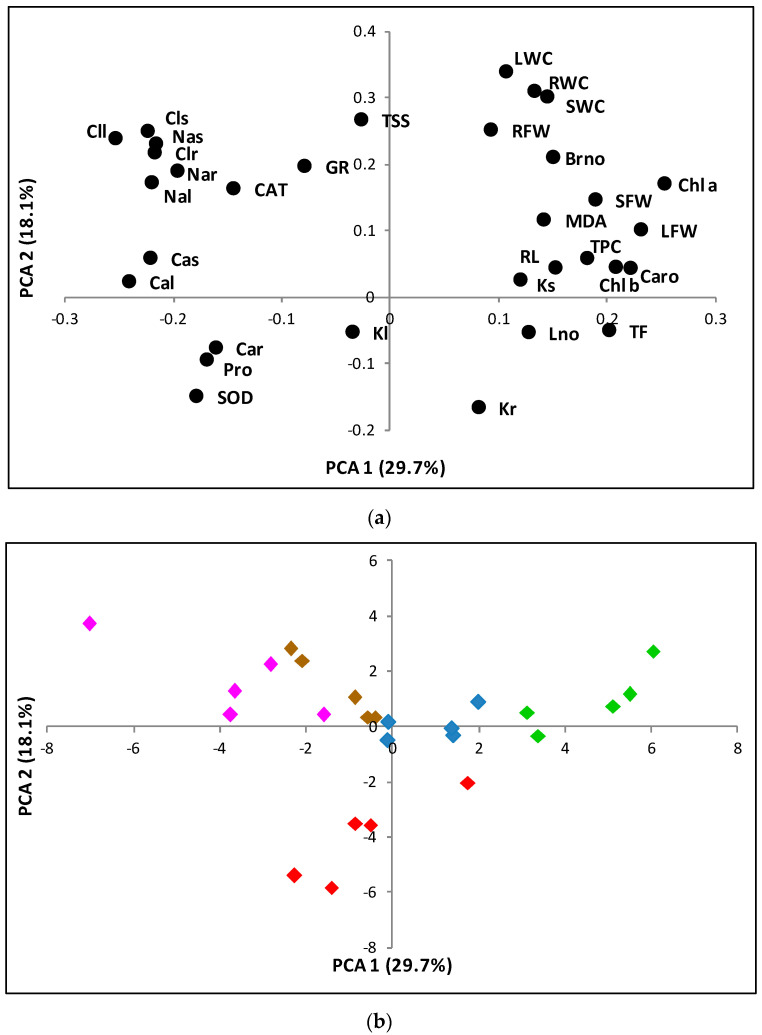
Loading plot (**a**) and scatterplot (**b**) of the principal component analysis (PCA) including all the analysed traits in *Thalictrum maritimum* plants subjected for 23 days to water deficit and salt treatments. The first (PC1; X-axis) and second (PC2; Y-axis) principal components accounted for 29.7% and 18.1% of the total variation, respectively. Abbreviations in the loading plot (**a**): root length (RL), root fresh weight (RFW), root water content (RWC), stem fresh weight (SFW), stem water content (SWC), branch number increment (Brno), leaf number increment (Lno), leaf fresh weight (LFW), leaf water content (LWC), chlorophyll a (Chla), chlorophyll b (Chlb), carotenoids (Caro), sodium in roots (Nar), sodium in leaves (Nal), potassium in roots (Kr), potassium in leaves (Kl), chloride in roots (Clr), chloride in leaves (Cll), proline (Pro), total soluble sugars (TSS), malondialdehyde (MDA), total phenolic compounds (TPC), total flavonoids (TF), and superoxide dismutase (SOD), catalase (CAT), and glutathione reductase (GR) activities. Symbols in the scatter plot (**b**): control (green), water stress (red), 100 mM NaCl (blue), 200 mM NaCl (brown), and 300 mM NaCl (pink).

A principal component analysis (PCA) was performed, including all analysed traits in all individuals grown in the greenhouse (Figure 8). Nine components with an Eigenvalue greater than one were identified, which overall explained 88.7% of the total variability. The first and second principal components accounted for 29.7% and 18.1% of the total variation, respectively. The first principal component showed positive correlations with growth parameters, such as the fresh weight of leaves (LFW) and stems (SFW); photosynthetic pigments: chlorophyll a (Chl a), chlorophyll b (Chl b), carotenoids (Caro); malondialdehyde (MDA), or total phenolic compounds (TFC)—all of which decreased under stress—and negative correlations with Na^+^ and Cl^−^ and catalase (CAT), which increased under salt stress. The second component was positively correlated with total soluble sugars (TSS) and water content of roots (RWC), stems (SWC) and leaves (LWC) and negatively correlated with K^+^ in leaves (Kl) and roots (Kr). (Figure 8a). 

The 25 individuals analysed were dispersed onto the two axes of the PCA scatterplot (Figure 8b), indicating a clear separation of the control, water stress and salt stress treatments. Plants from the control and salt treatments were distributed along the X-axis, from higher positive values (non-stressed controls) to higher negative values (300 mM NaCl), whereas those from the water stress treatment were located along the second principal component (Y-axis).

## 3. Discussion

*Thalictrum maritimum* is a rare endemic, found only in several salt marshes near Valencia in Eastern Spain. In the Natural Park of Albufera, where the species was first described, its distribution is not continuous; the plants appear spread through small populations in different salt marshes, known locally as ‘malladas’, occupying the inter-dune depressions. The most abundant population is that of ‘Mallada del Canyar’, where the soil samples were collected. As stated in the Introduction, the change of the populations in the park was followed between 2011 and 2017, reaching a maximum in 2015, but decreasing in 2017, the year of the last census, characterised by very dry summer and autumn (Servicio de Vida Silvestre, unpublished data). Dry summers represent a key trait of the Mediterranean climate, which is characterised by a pronounced water deficit in summer, often with periods of more than one month without registering any rainfall, while precipitation is concentrated in autumn and spring [23]. The decrease in almost 1000 adult individuals from 2015 to 2017 is probably related to the exceptionally dry autumn in the second year. A wet autumn is necessary for a good regeneration of rhizomes in geophytes such as *T. maritimum,* which spend a large part of the year underground. The survey performed in this study, even though it did not include an exhaustive census, revealed that several of the small populations had disappeared, despite the conservation efforts undertaken. The climatic analysis indicated an increment of the summer temperature in the last few years and a reduction of precipitation, which may also be related to this reduction. 

The range of tolerance of *T. maritimum* was checked by applying water deficit and salt stress treatments under controlled greenhouse conditions. The results revealed that the species’ tolerance to salinity is far beyond that registered under natural conditions. Soil salinity, analysed in all locations where the species was present, was generally low, as previously reported in a study on soils characteristics in the habitats with *T. maritimum* and other Valencian endemics [25]; in some locations, soil was not saline at all. The vegetation accompanying *T. maritimum* includes several halophytic species, such as *Plantago crassifolia, Schoenus nigricans, Juncus acutus* and *J. maritimus*, but also glycophytes, specific of communities developed on non-saline soils like those of the tall shrublands and pinewood vegetation of the *Quercetea ilicis* phytosociological class; for example, *Phillyrea angustifolia, Pistacia lentiscus* and *Smilax aspera.* Important soil parameters related to the distribution of *T. maritimum* are moisture and organic matter content. Registered soil moisture values were above those reported in other studies in the same area [26,27]. The percentage of organic matter in *T. maritimum* locations was also higher than that reported for soils with sand texture from other salt marshes in the Natural Park of Albufera [28]. 

Under greenhouse controlled conditions, *T. maritimum* proved to be more salt-tolerant than expected. Plants survived for more than three weeks at concentrations of 300 mM NaCl; the progressive accumulation of salt generated in the pot substrate an EC of 18 dS m^−1^ at the end of the treatment, substantially higher than that registered in the field. This strong saline treatment, however, was not harmless to the plants since the measurement of different morphological parameters showed inhibition of growth with respect to non-stressed controls. At lower salinity (100 mM NaCl, ca. 10 dS m^−1^ after 23 days of treatment), no significant changes were detected in the analysed growth traits, even though the substrate EC was still much higher than in most of the studied natural locations of the species. Nevertheless, to compare the effects of water deficit and salinity, all treatments had to be stopped after 23 days, due to the strong negative impact of water stress, as plants which did not receive irrigation showed intense wilting. Degradation of photosynthetic pigments was observed in all stress treatments with respect to controls, but the reduction of chlorophylls and carotenoids contents was more pronounced in plants subjected to the water stress treatment.

Therefore, we may conclude that, under the specific conditions of our experiments, water stress has a stronger negative effect on *T. maritimum* than salinity. The severe water deficit treatment applied to the plants in the greenhouse may mimic the conditions of the long and intense drought periods affecting the plants in their natural habitats during summer. In contrast, even though *T. maritimum* is present in low-salinity zones of the salt marshes, the biochemical analysis of its responses to salt stress highlighted that the species has many attributes of typical halophytes; most important, its primary mechanism of tolerance appears to be based on the active transport of toxic ions to the aboveground organs of the plants. The concentrations of Na^+^ and Cl^−^ increased in all organs in parallel to the increase of the concentration of NaCl in the irrigation solution, but the ions accumulated mostly in the foliar tissue, as reported in many salt-tolerant plants (e.g., [28,29,30]). Accumulation of inorganic ions as ‘cheap’ osmotica (in terms of energy consumption), is a widespread strategy of tolerance in halophytes [31,32]. According to the ‘ion compartmentalisation hypothesis’ [33,34], Na^+^ and Cl^−^ should be predominantly stored in leaf vacuoles, to avoid reaching toxic concentrations in the cytosol.

As Na^+^ and K^+^ compete for the same binding sites in proteins, including membrane transporters, accumulation of Na^+^ is generally associated with a drop in cellular K^+^ levels; furthermore, plasma membrane depolarisation, triggered by high Na^+^ concentrations, also causes the loss of cellular K^+^ by activation of outward rectifying K^+^ channels [35]. In the present study, the homeostasis of K^+^ was maintained under both types of stress, as no significant differences in K^+^ concentration with respect to the corresponding controls were registered in salt and water-stressed plants. The levels of K^+^ in stems and leaves were higher than in roots under all treatments, indicating the presence of active transport of K^+^ from the roots to the shoots of the plants. This mechanism partly counteracts the harmful effects of Na^+^, enabling the maintenance of high leaf levels of K^+^, necessary for the metabolic processes [36]; this is a common feature of many halophytes, although it has also been reported for some glycophytes [26,37].

A significant increase in Ca^2+^ concentration in roots, stems and leaves was observed in the plants, in response to the water deficit and salt stress treatments. The protective effects of calcium against salt stress and sodium toxicity have been long known [38,39]. Calcium is essential for plant growth under stress conditions, playing regulatory and signalling roles, for example, maintaining Na^+^ and K^+^ homeostasis via the SOS pathway [40,41]. Therefore, Ca^2+^ uptake and accumulation may contribute to tolerance to stress in *T. maritimum*, not only to high salinity but also to water deficit. Indeed, increased concentrations of Ca^2+^ have been reported in several typical halophytes, such as species of the genus *Limonium*, both under salt stress [30] and under water stress [42]. As observed for the other analysed ions, higher Ca^2+^ concentrations have been measured in the aboveground organs of the plants than in the roots, under high salinity conditions (but not in response to water stress).

The accumulation of inorganic ions is balanced by the accumulation of compatible solutes, or osmolytes, which not only contribute to cellular osmotic adjustment under stress but are also involved in other mechanisms of stress tolerance, acting as low-molecular-weight chaperones, reactive oxygen species (ROS) scavengers or signalling molecules [43,44,45]. Proline (Pro) is one of the most common osmolytes in plants, accumulating to high levels in many species in response to different abiotic stresses [46]. In our experiments, leaf Pro contents increased in water- and salt-stressed plants, but the increase over control values and the absolute Pro concentrations reached were too low to have any relevant osmotic effect. Nevertheless, a contribution of Pro to stress tolerance in *T. maritimum*, based on its ‘osmoprotectant’ or signalling functions, cannot be ruled out. TSS contents did not show significant changes in response to the salt treatments, and only a slight (but significant) decrease in the water-stressed plants, suggesting that these compounds are also not involved in osmotic adjustment in *T. maritimum*. Probably, this function is mostly fulfilled by the stress-induced accumulation of inorganic ions, without excluding the possibility that some additional organic osmolyte, not identified in the present work, contributes to osmotic balance under stress in this species. 

Salinity and drought are usually associated with oxidative stress, due to excessive accumulation of reactive oxygen species (ROS). Small amounts of ROS are generated during normal metabolic processes, such as photorespiration, photosynthesis and respiration, and play an essential role as signalling molecules [47,48]. ROS production, however, is considerably increased during abiotic stress [49,50,51]; when in excess, ROS generate major metabolic disturbances that can lead to cell death [52,53,54,55]. Excessive ROS accumulation is prevented or limited by the synthesis of antioxidant compounds and the activation of antioxidant enzymatic systems. Phenolic compounds and, particularly, the subgroup of flavonoids, are strong antioxidants in plants [47], whereas some of the most common antioxidant enzymes include superoxide dismutase (SOD), catalase (CAT), or redox regulatory enzymes such as glutathione reductase (GR), among many others [56]. At the cellular level, SOD represents the first line of defence against ROS [47], catalysing the dismutation of the superoxide radical to oxygen and H_2_O_2_ [57]. SOD has been found in all aerobic organisms examined to date [58] and is generally activated in plants in response to different environmental stress signals, although there are also reports showing contradictory results [59]. CAT acts after SOD, catalysing the elimination of H_2_O_2_, which is transformed into water and oxygen [58].

MDA is a final product of polyunsaturated fatty acids peroxidation and is widely used as a marker of oxidative stress [60]. Therefore, an increase in MDA levels should be expected in plants that are subjected to different stress treatments, and this has indeed been reported for many species [61,62,63]. In the present study, however, MDA contents did now show any significant change in response to water deficit or high salinity, indicating that, under the specific conditions of our experiments, high levels of oxidative stress were not generated. The lack of secondary oxidative stress associated with salt and water stress is not common in stress-sensitive plants but has been previously reported in many halophytes, both in their natural habitats [51,64,65] and under controlled greenhouse conditions [42]. In *T. maritimum*, redox equilibrium seemed to be maintained by a slight increase in the specific activity of SOD, under conditions of water deficit and high salinity, whereas activation of CAT and GR, also weak, was only detected in the presence of high salt concentrations. No additional contribution of antioxidant compounds was necessary and, consequently, we did not notice any increase in the concentrations of TPC or TF in the stressed plants.

Caution should be taken when attempting to extend the results obtained under controlled greenhouse conditions to the behaviour of the plants in the field. First, it is not possible to directly compare their responses to stress when growing in the artificial environment of a pot, or in their natural salt marsh habitat. On the other hand, plants in nature are simultaneously affected by different environmental stress conditions, which may interact in complex ways; for example, the responses to drought and soil salinity in the field cannot be considered independently of one each other, as we have done in the greenhouse experiments. Notwithstanding these limitations, we believe that the results presented here can guide the design and implementation of conservation programmes for this endangered species, helping to find the optimal sites for reintroductions or translocations from threatened areas. *Thalictrum maritimum* could grow in zones of high salinity within the salt marshes, unless it encounters strong competition from more abundant, structural halophytes. However, some local sites in the Albufera Natural Park, where *T. maritimum* vanished in the past, may not be the best ones to perform reintroduction projects, e.g., if the sites have been excessively clogged by sediments and not enough water level can be ensured. On the contrary, maybe sites closer to the Albufera lake, where the underground water table is more stable, and higher soil moisture can be maintained, could be recommended for future translocation trials.

## 4. Materials and Methods

### 4.1. Study Area

The area of study is known as ‘Devesa de l’Albufera’, a close to 10 km long dune system, occupying 800 ha and belonging to the municipality of Valencia, in eastern Spain. This large dune complex is a sandy coastal strip that progressively closed an ancient maritime bay, nowadays converted into the Albufera lake. The Albufera, covering ca. 2000 ha, is the biggest continental lake in Spain, as well as the second most important Spanish wetland [11]. Both, sand coastal dunes and the Albufera lake, as well as a large belt of traditional rice fields, are part of the Albufera Natural Park, officially declared in 1986 by the Valencian government. This natural park covers a surface of 21,000 ha. It is the most celebrated protected natural area in the Valencian region and one of the most important in Western Europe, mainly regarding waterfowl migrations [11]. Since 1990, the site has been included in the list of Wetlands of International Importance of the Ramsar Convention; in 1991, it was declared a Special Protection Area under the EU Directive on the Conservation of Wild Birds (79/409/EEC). In addition, the park includes habitats and refuges of species included in the EU Habitats Directive (92/43/EEC) and is also included in the Geneva Protocol on Special Protection Areas in the Mediterranean [66].

The populations of *Thalictrum maritimum* live in the so-called ‘malladas’, inter-dune depressions located in the Devesa area, acting as small salt marshes, often inundated during the winter period.

### 4.2. Habitat Survey

A first survey was carried out in situ to verify the existence or disappearance of the populations collected in the former literature [10,67] and official records on the species distribution in the natural park, kindly provided by the regional Wildlife Service (Servicio de Vida Silvestre) and the municipal Devesa-Albufera Service. 

For the sites where *T. maritimum* is currently extant, vegetation relevés were carried out on each population site. Geographic location was obtained with a Garmin GPS in UTM coordinates (ETRS 89, zone 30); they have not necessarily been taken in the centre of the relevé to avoid trampling, which is aggravated when the soil is waterlogged. The phytosociological method was used to note the proportions in which the species appear [20]. Nomenclature of the taxa follows Euro Med [68] and the syntaxonomic nomenclature is according to Rivas-Martinez et al. [21,22]. In each relevé site, three measurements of soil parameters were taken: moisture (%), electric conductivity (dS m^−1^) and temperature (°C), with a WET sensor (Delta Devices, Cambridge, UK). The relevés were carried out during the optimal phenological time, mainly from mid-June to mid-November 2019, because this is the time when *T. maritimum* individuals are easily detected; besides, this is the more recommended time to avoid disturbance to wintering birds, which are the most relevant protection object of the natural park [11].

### 4.3. Climatic Analysis

Climate data were retrieved from SIAR, the Agroclimatic Information System for Irrigation [24] of the Spanish Ministry of Agriculture, Fisheries and Food. Data on the mean, maximum and minimum temperatures, rainfall and reference evapotranspiration (ETo) were collected for the past 19 years, on a monthly basis, from the agroclimatological station Benifaio, located at 11 km from the area of study. References to bioclimatic classification were made according to the Worldwide Bioclimatic Classification System [23].

### 4.4. Soil Analyses

For a complete analysis, eight soils samples were taken from the salt marsh where the most abundant population of *T. maritimum* was found (‘Mallada del Canyar’), four at 0–10 cm and four at 10–20 cm depth. The samples were air-dried at room temperature, and then crushed with a roller to break aggregates, and passed through a 2-mm sieve. Analyses were performed on fine soil (diameter <2 mm). Soil texture was analysed by the hydrometer method [69]. Organic matter was determined by the Walkley and Black method [70] and carbonates by using the Bernard calcimeter [71]. The following parameters were analysed in a saturation extract: pH, electric conductivity (EC), chlorides, Na^+^, K^+^, Ca^2+^, and Mg^2+^. A Crison pH-meter Basic 20 and a Crison Conductimeter Basic 30 (Crison Instruments SA, Barcelona, Spain) were used to measure pH and EC, respectively. Sodium and potassium were quantified with a PFP7 flame photometer (Jenway Inc., Burlington, VT, USA), chlorides were measured in an MKII Chloride Analyzer 926 (Sherwood, Inc., Cambridge, UK), and divalent cations, Ca^2+^ and Mg^2+^, were measured with an atomic absorption spectrometer SpectrA 220 (Varian, Inc., Palo Alto, CA, USA).

### 4.5. Plant Growth and Stress Treatments in the Greenhouse

Young plants of *T. maritimum*, provided by the ‘Centre for Conservation of Freshwater Species of the Valencian Community’, were transplanted individually to standard 1.6 L plastic pots, in a mixture of peat, perlite and vermiculite (2:1:1), and maintained in the greenhouse under a 12 h-light/12 h-dark photoperiod, at 23 °C during the day and 18 °C during the night. The stress treatments were initiated after three weeks of acclimatisation. The pots were divided into five batches (one per treatment) of five pots each and placed in plastic trays (55 × 40 cm), and the following treatments were applied: control (C), water stress (WS), salt treatments (100 mM, 200 mM and 300 mM NaCl), over a period of 23 days. Plants subjected to the WS treatment were not irrigated at all during this period, whereas the remaining plants were irrigated twice a week by adding to each tray 1.5 L of deionised water (for the control treatment) or NaCl aqueous solutions of the final concentrations indicated above (for the salt treatments); in the latter case, the trays were thoroughly washed with deionised water before addition of the NaCl solutions. The treatments were stopped after 23 days, when plants subjected to the water deficit treatment showed intense wilting, but before mortality was observed.

### 4.6. Substrate Analysis

Soil moisture, expressed as volume percentages, and soil electroconductivity (EC), expressed in dS m^−^¹, were determined in the pots substrate at the beginning, during and at the end of the treatments, with a WET-2 Sensor (Delta—T Devices, Cambridge, UK).

### 4.7. Plant Growth Parameters

Non-destructive plant growth parameters, such as stem length, number of leaves and number of branches, were determined at the beginning and the end of the treatments. Fresh weight of leaves, stems and roots were separately measured after 23 days of treatment, when all plants were sampled. Part of the fresh material of each organ was weighed (fresh weight; FW), dried for four days at 65 °C, until constant weight, and then weighed again (dry weight; DW), to calculate the water content percentage (WC%) for roots, stems and leaves, according to the following formula: [65].

WC% = [(FW − DW)/FW] × 100(1)

### 4.8. Photosynthetic Pigments

Chlorophylls a and b (Chl a, Chl b) and total carotenoids (Caro) were determined as previously described [72]. Ten mL of ice-cold 80% (*v/v*) acetone was used to extract pigments from 0.05 g of fresh leaf material. After mixing overnight and centrifuging for 10 min at 12,000 rpm, the supernatant was collected, and its absorbance was measured at 663, 646, and 470 nm. Chl a, Chl b, and Caro concentrations were calculated using the equations below, described by Lichtenthaler et al. [72].

Chlorophyll a (µg ml^−1^) = 12.21 × A_663_ − 2.81 × A_646_ (2)

Chlorophyll b (µg ml^−1^) = 20.13 × A_646_ − 5.03 × A_663_ (3)

Carotenoids (µg ml^−1^) = (1000 × A_470_ − 3.27 × [Chlorophyll a] − 104 × [Chlorophyll b])/227 (4)

Pigment contents were finally expressed in mg g^−1^ DW.

### 4.9. Ion Quantification

The content of Na⁺, K⁺ and Ca²⁺ was quantified separately from roots, stems and leaves, according to the protocol described by Weimberg [73], in aqueous extracts of dry plant material. Two mL of Milli-Q water were added to each sample (0.1 g), vortexed and then mixed for 24 h in a shaker. The samples were incubated in a water bath for 30 min at 95 °C, cooled on ice, and filtered through a 0.45 µm nylon filter. Sodium, potassium and calcium ions were quantified using a PFP7 flame photometer (Jenway Inc., Burlington, VT, USA), and chlorides were measured in an MKII Chloride Analyzer 926 (Sherwood, Inc., Cambridge, UK). 

### 4.10. Osmolyte Quantification

Proline (Pro) contents were measured according to the ninhydrin-acetic acid method [74]. Extracts were prepared by grinding 0.1 g of fresh leaf material in two mL of a 3% (*w/v*) sulfosalicylic acid solution; the samples were mixed with acid ninhydrin, placed in a water bath at 95 °C for one hour, cooled on ice for 10 min, and extracted with toluene. The absorbance of the organic phase was measured at 520 nm, using toluene as the blank. A standard curve was obtained running parallel reactions containing known Pro concentrations. Leaf Pro contents were finally expressed in µmol g^–1^ DW.

Total soluble sugars (TSS) were quantified according to the protocol described by Dubois et al. [75]. Fresh leaf material (0.1 g) was ground and extracted with two mL of 80% (*v/v*) methanol, by mixing the samples in a shaker for 24 h, followed by centrifugation at 12,000 rpm for 15 min. Subsequently, each supernatant, appropriately diluted with water, was mixed with 95% sulphuric acid and 5% phenol. Finally, the absorbance of the samples was measured at 490 nm. TSS concentrations were expressed as mg equivalent of glucose, used as the standard (mg eq. Gluc g^−1^ DW).

### 4.11. Oxidative Stress Markers and Non-Enzymatic Antioxidants

Malondialdehyde (MDA), total phenolic compounds (TPC) and total flavonoids (TF) were measured in the same 80% methanol extracts used for TSS determination. MDA contents were quantified as previously described by Hodges et al. [76], with some modifications [77]. The methanol extracts were mixed with 0.5% (*w/v*) thiobarbituric acid (TBA) prepared in 20% (*w/v*) trichloroacetic acid (TCA). The samples were incubated in a water bath at 95 °C for 15 min, cooled on ice for five min and centrifuged at 12,000 rpm for 10 min at 4 °C; the absorbance of the supernatants was measured at 532 nm. After subtracting the non-specific absorbance at 440 nm and 600 nm, MDA concentrations in the extracts were calculated using the equation included in Taulavuori et al. [77], based on the extinction coefficient of the MDA-TBA adduct at 532 nm (155 mM^−1^ cm^−1^). Extracts mixed with TCA but without TBA, were assayed in parallel and used as controls. MDA contents were finally expressed as nmol g^−1^ DW.

Total phenolic compounds (TPC) were quantified by reaction with the Folin-Ciocalteu reagent, as previously described [78]. Leaf methanol extracts were mixed with the reagent and 15% (*w/v*) Na₂C0₃ and incubated at room temperature for 90 min in the dark. Finally, the absorbance of the samples was measured at 765 nm. A calibration curve was obtained from samples containing known amounts of gallic acid (GA), assayed in parallel. TPC concentrations were expressed as equivalents of gallic acid (mg eq. GA g^−1^ DW).

Total flavonoids (TF) were determined by the method described by Zhishen et al. [79], based on the nitration with NaNO_2_ of aromatic rings containing a catechol group, followed by reaction with AlCl_3_ at a basic pH. After the reaction, the absorbance of the samples was determined at 510 nm. TFs concentrations were expressed as equivalents of catechin, used as the standard (mg eq. C g^−1^ DW). 

### 4.12. Antioxidant Enzymes Assays

Enzyme specific activities were determined in crude protein extracts, prepared from leaf material stored frozen at −75 °C, as previously described [65]. The protein concentration in the extracts was determined by the Bradford method [80], using bovine serum albumin (BSA) as the standard and the Bio-Rad reagent (Bio-Rad Laboratories, Alcobendas, Spain).

Superoxide dismutase (SOD) activity was assayed following the procedure described by Beyer and Fridovich [81], by monitoring the inhibition of nitroblue tetrazolium (NBT) photoreduction in reaction mixtures containing riboflavin as the source of superoxide radicals. After the addition of riboflavin and NBT, the samples were irradiated with 15 W fluorescent tubes for 10 min at 25 °C, and the absorbance at 560 nm was measured. One SOD unit was defined as the amount of enzyme causing a 50% inhibition of NBT photoreduction under the assay conditions.

Catalase activity (CAT) was determined as described by Aebi [82]. The assay is based on the consumption of H₂O₂ added to the reaction mixtures by the catalase present in the extracts, which is followed by the decrease in absorbance at 240 nm. One CAT unit was defined as the amount of enzyme that breaks down one mmol of H₂O₂ per minute at 25 °C.

Glutathione reductase (GR) activity was quantified by the decrease in absorbance at 340 nm due to the oxidation of NADPH, the cofactor in the GR-catalysed reaction of reduction of oxidised glutathione (GSSG) to reduced glutathione (GSH). One GR unit was defined as the amount of enzyme necessary to oxidise one mmol of NADPH per minute at 25 °C [83].

### 4.13. Statistical Analysis

Data were analysed using the programme Statgraphics Centurion XVII (Statgraphics Technologies, The Plains, VA, USA). All mean values throughout the text are based on five biological replicates. Significant differences between treatments were tested by one-way analysis of variance (ANOVA) at the 95% confidence level, and post hoc comparisons were made using the Tukey’s HSD test at *p* ≤ 0.05. A principal component analysis (PCA) was used to check the similarity between the responses to water and salt stress.

## 5. Conclusions

*Thalictrum maritimum* is a moderate halophytic species, with optimal growth in the absence of salt, but tolerating concentrations of more than 300 mM NaCl. The primary mechanism of salt tolerance appears to be related to the active transport of ions to the aboveground organs of the plants and the maintenance of high K^+^ contents with increasing Na^+^ concentrations. However, the species is more sensitive to water deficit, as indicated by field and greenhouse analyses. The survival of this rare species is not threatened by increased soil salinisation in the salt marshes where it is present, an expected effect of climate change, as greenhouse experiments indicated that *T. maritimum* tolerates salinity well beyond that registered in its natural environment. However, water stress, which can also increase in the salt marsh habitat as a consequence of global warming, may endanger the populations of *T. maritimum*. Both the analyses of the changes in its populations in relation to climatic factors in the area of study and the water stress treatments performed in the greenhouse indicated that the most harmful abiotic stress factor for this species is represented by drought. 

## Figures and Tables

**Table 1:** Phytosociological relevés with *Thalictrum maritimum* from the Natural Park of Albufera. The extension and vegetation coverage, as well as soil properties (moisture and electric conductivity) are shown for each of the ten relevés. Ranges of cover of the species present in each relevé are indicated, according to the Braun-Blanquet’s cover-abundance scale [20]: occasional presence, <5% (+); 5–12% (**1**); 12–25% (**2**); 25–50% (**3**); 50–75% (**4**); 75–100% (**5**). The species are classified into their corresponding vegetation classes [21,22].

Relevé nº		1	2	3	4	5	6	7	8	9	10
Area (m^2^)		25	100	90	100	8	100	100	60	100	100
Vegetation coverage (%)		100	95	100	100	100	100	100	100	100	100
Moisture (%)		40.2	59.4	60.5	58.3	32.0	50.7	39.9	20.6	56.3	51.9
Conductivity (EC, dS m^−1^)		3.7	2.1	1.9	2.1	12.2	3.8	1.3	3.0	1.6	2.4
**Species**	**Vegetation Class**										
	*Juncetea maritimi*										
*Thalictrum maritimum*		3	3	+	3	1	1	2	2	4	2
*Centaurea dracunculifolia*					2			+			1
*Dorycnium gracile*			+				1	2			2
*Elymus elongatus*								1			+
*Juncus acutus*						2					
*Juncus maritimus*		2	2	3	2			1	1	1	
*Linum maritimum*		+	+	1	+			1			
*Plantago crassifolia*		+				1					
*Samolus valerandi*		+	+	1							
*Schoenus nigricans*		3	2	2	1	2		2		1	2
*Scirpioides holoschoenus*						2					
*Spartina patens*						3	5	3			2
	*Phragmito-Magnocaricetea*										
*Cladium mariscus*							+	+	4	3	2
*Lythrum salicaria*									1	1	1
*Phragmites australis* subsp. *hrysanthus*		3	3	4	1		+	+	1	2	1
*Phragmites australis* subsp. *australis*		+	+	+	1					1	
	*Molinio-Arrhenatheretea*										
*Sonchus maritimus*		+	1	1	1						
	*Nerio-Tamaricetea*										
*Tripidium ravennae*		+	+	+	1				1		+
*Imperata cylindrica*											+
	*Quercetea ilicis*										
*Phillyrea angustifolia*			+		+						
*Pistacia lentiscus*								+	+	1	1
*Smilax aspera*								+			
	*Stellarietea mediae*										
*Lagurus ovatus*		1	+	+	+				+		

In addition: Artemisetea vulgaris: Ditrichia viscosa in **3**, **4**, **5**; Galio-Urticetea: Cynanchum acutum in **3**, **4**; Salicornietea fruticosae: Inula crithmoides in **1**; Thero-Salicornietea: Centaurium spicatum, Suaeda spicata in **1**.

**Table 2:** Plant communities identified in the phytosociological analysis.

Communities	Relevé nº
*Juncetalia maritimi*	1, 2, 3, 4
*Hydrocotylo-Mariscetum serrati*	8, 9, 10
*Spartino-Juncetum maritimi* subass. *spartinetosum*	5, 6, 7

**Table 3:** Meteorological data for the last 19 years obtained from the agroclimatological station Benifaio; data provided by the Agroclimatic Information System for Irrigation (SIAR) [24]. T: temperature; Hum: humidity; Eto: evapotranspiration.

Year	Mean T (°C)	Max T (°C)	Min T (°C)	Mean Hum (%)	Max Hum (%)	Min Hum (%)	Rainfall (mm)	Eto (mm)
2001	17.98	29.24	7.88	65.38	93.35	19.41	313.80	1119.18
2002	17.44	29.09	7.43	67.93	94.27	21.22	322.70	1091.91
2003	17.60	29.47	6.54	67.65	93.35	18.84	289.00	1155.10
2004	17.63	28.95	7.13	69.57	96.33	18.40	594.40	1006.45
2005	16.45	29.50	4.48	68.32	95.89	16.36	377.60	1117.97
2006	17.53	29.04	6.93	69.13	95.68	18.00	464.40	1189.38
2007	16.81	29.60	5.90	68.13	95.31	16.26	894.40	1164.50
2008	16.88	29.45	5.91	68.35	95.67	17.74	674.40	1194.10
2009	17.34	29.85	6.26	68.60	97.16	19.03	446.20	1215.26
2010	16.78	29.69	5.54	68.31	97.06	19.32	565.00	1206.22
2011	17.57	30.45	6.87	70.32	96.52	18.75	472.00	1166.73
2012	17.31	30.46	5.13	67.58	98.34	18.41	503.61	1208.25
2013	17.55	29.92	6.23	63.26	95.27	16.95	263.80	1245.42
2014	18.32	30.81	8.02	65.32	95.90	15.54	224.40	1278.22
2015	17.76	30.88	7.10	70.02	98.56	17.34	401.26	1169.08
2016	17.85	29.66	6.46	68.66	98.42	20.57	259.57	1218.41
2017	17.59	29.97	6.77	68.51	97.63	18.44	307.26	1238.82
2018	17.60	29.55	6.85	68.06	97.09	22.58	684.02	1225.71
2019	17.79	31.41	7.25	66.59	97.58	19.49	427.00	1243.83
Mean	17.46	29.84	6.56	67.88	96.28	18.56	446.57	1181.82

**Table 4:** Soil characteristics in the salt marsh ‘Mallada del Canyar’, where the most abundant population of *T. maritimum* is located. Values represent means followed by SE (*n* = 4).

Parameter	0–10 cm Depth	10–20 cm Depth
Sand (%)	95.00 ± 0.28	93.56 ± 1.05
Silt (%)	3.50 ± 0.19	4.49 ± 0.74
Clay (%)	1.49 ± 0.08	1.92 ± 0.31
Apparent density (g cm^−3^)	1.09 ± 0.09	1.15 ± 0.07
Porosity (%)	58.86 ± 3.82	56.60 ± 2.64
Carbonates (%)	22.51 ± 4.69	28.23 ± 9.58
Organic Matter (%)	1.96 ± 0.75	1.29 ± 0.21
pH	7.30 ± 0.30	7.39 ± 0.22
EC (dS m^−1^)	5.01 ± 3.45	3.06 ± 1.32
Na^+^ (meq L^−1^)	46.36 ± 6.77	42.67 ± 4.62
K^+^ (meq L^−1^)	1.55 ± 0.56	1.26 ± 0.22
Cl^−^ (meq L^−1^)	20.55 ±5.37	28.18 ± 2.22
Ca^2+^ (meq L^−1^)	7.51 ± 2.21	6.43 ± 0.70
Mg^2+^ (meq L^−1^)	5.48 ± 3.32	3.93 ± 0.81

**Table 5:** Leaf concentrations of proline (Pro) and total soluble sugars (TSS), malondialdehyde (MDA), total phenolics (TPC) and total flavonoids (TF), and specific activities of antioxidant enzymes, superoxide dismutase (SOD), catalase (CAT) and glutathione reductase (GR), after 23 days of stress treatments of *Thalictrum maritimum* plants grown in controlled conditions in the greenhouse. Values shown are means ± SE (*n* = 5). Same lowercase letters in each row indicate homogeneous groups between treatments according to the Tukey test (*p* ≤ 0.05).

Biochemical Trait	Control	Water Stress	100 mM NaCl	200 mM NaCl	300 mM NaCl
Pro (µmol g^−1^ DW)	13.3 ± 1.2 ^a^	20.7 ± 2.6 ^b^	20.7 ± 2.5 ^b^	15.9 ± 0.8 ^ab^	20.5 ± 2.1 ^b^
TSS (mg eq. Gluc g^−1^ DW)	352.2 ± 80.7 ^bc^	233.1 ± 37.9 ^a^	265.5 ± 6.0 ^ab^	418.7 ± 46.0 ^c^	401.0 ± 38.5 ^bc^
MDA (nmol g^−1^ DW)	365.1 ± 52.9 ^a^	268.6 ± 50.7 ^a^	323.4 ± 28.2 ^a^	296.1 ± 23.5 ^a^	255.0 ± 16.7 ^a^
TPC (mg eq GA g^−1^ DW)	26.6 ± 5.6 ^b^	21.1 ± 2.1 ^ab^	19.0 ± 1.4 ^ab^	19.9 ± 2.2 ^ab^	17.5 ± 1.7 ^a^
TF (mg eq C g^−1^ DW)	10.7 ± 1.5 ^c^	8.1 ± 1.7 ^bc^	5.8 ± 0.6 ^ab^	4.0 ± 1.2 ^a^	4.1 ± 0.2 ^a^
SOD (U g^−1^ protein)	13.2 ± 1.2 ^a^	46.9 ± 8.8 ^c^	23.6 ± 5.4 ^a^	33.0 ± 3.6 ^b^	30.3 ± 4.1 ^b^
CAT (U g^−1^ protein)	228.6 ± 26.4 ^ab^	158.3 ± 87.3 ^a^	314.6 ± 26.9 ^b^	272.8 ± 33.3 ^ab^	544.5 ± 31.9 ^c^
GR (U. g^−1^ protein)	1543.9 ± 342.0 ^a^	1057.5 ± 249.0 ^a^	1624.9 ± 330.0 ^a^	2854.5 ± 137.0 ^b^	1793.9 ± 354.0 ^a^
